# Sphingolipid Levels and Signaling via Resveratrol and Antioxidant Actions in Cardiometabolic Risk and Disease

**DOI:** 10.3390/antiox12051102

**Published:** 2023-05-16

**Authors:** Melania Gaggini, Simona Fenizia, Cristina Vassalle

**Affiliations:** 1 Institute of Clinical Physiology, National Research Council of Italy (CNR), Via Moruzzi 1, I-56124 Pisa, Italy; melania.gaggini@ifc.cnr.it (M.G.); simona.fenizia@ifc.cnr.it (S.F.); 2 Fondazione G. Monasterio CNR-Regione Toscana, Via Moruzzi 1, I-56124 Pisa, Italy

**Keywords:** resveratrol, sphingolipids, ceramides, sphingosine-1-phosphate, cardiometabolic risk, cardiometabolic disease

## Abstract

Resveratrol (RSV) is a phenolic compound with strong antioxidant activity, which is generally associated with the beneficial effects of wine on human health. All resveratrol-mediated benefits exerted on different systems and pathophysiological conditions are possible through resveratrol’s interactions with different biological targets, along with its involvement in several key cellular pathways affecting cardiometabolic (CM) health. With regard to its role in oxidative stress, RSV exerts its antioxidant activity not only as a free radical scavenger but also by increasing the activity of antioxidant enzymes and regulating redox genes, nitric oxide bioavailability and mitochondrial function. Moreover, several studies have demonstrated that some RSV effects are mediated by changes in sphingolipids, a class of biolipids involved in a number of cellular functions (e.g., apoptosis, cell proliferation, oxidative stress and inflammation) that have attracted interest as emerging critical determinants of CM risk and disease. Accordingly, this review aimed to discuss the available data regarding the effects of RSV on sphingolipid metabolism and signaling in CM risk and disease, focusing on oxidative stress/inflammatory-related aspects, and the clinical implications of this relationship.

## 1. Introduction

Resveratrol (RSV) is a phenolic compound with highly significant antioxidant properties, which are often associated with the beneficial effects of wine on human health, as it is contained at high percentages in red wine. Although the mechanisms of action of RSV are complex and pleiotropic, it is clear that its antioxidant properties substantially contribute to the beneficial effects of this molecule. Most of the current knowledge on the activity and effects of RSV is derived from in vitro, ex vivo and animal studies, whereas evidence obtained from humans is still affected by short-term interventions, which have often been performed on a small number of subjects [1]. Additional information has been derived from Mediterranean diet cohorts, which support the beneficial role of polyphenol-rich foods (which is clearly also valid for wine and RSV) in cardiometabolic (CM) health [2,3]. Nevertheless, the evaluation of specific, single components of the Mediterranean diet (as RSV) remains challenging owing to the fact that wine (the main source of RSV, not accounting for the entire dietary pattern) is also rich in many other bioactive substances (e.g., other polyphenolic compounds such as quercetin, catechin, epicatechin and anthocyanin). All these natural components interact both with each other (either increasing or decreasing the effect) and with many other confounders of a different nature other than diet (e.g., physical and outdoor activities), which ultimately contribute to the final balance between beneficial or adverse effects on CM health [4].

The way in which polyphenols are consumed should also be considered. Indeed, while experimental data show, on the one hand, several positive effects of RSV in mice (e.g., reduced albuminuria, decreased inflammation and apoptosis on the vascular endothelium level, increased aortic elasticity, greater motor coordination, reduced cataract formation and the maintenance of bone mineral density), on the other hand, these effects were not observed when RSV was given to mid-life mice (older than 12 months of age) [5]. Moreover, recent results indicate that different isoforms of RSV (cis or trans) exert opposite physiological effects [6]. Nonetheless, RSV’s anti-cancer, anti-aging and anti-inflammatory/antioxidant actions, as well as its cardiometabolic and neuronal-protective effects, have been widely demonstrated [7]. In particular, experimental results highlight a number of RSV benefits on CM risk and disease (e.g., the improvement of the lipid profile, oxidative stress reduction, the prevention of platelet aggregation and the maintenance of endothelial function through the enhancement of nitric oxide (NO) bioavailability and inhibition of endothelin (ET-1)) [8,9,10,11]. In humans, RSV has been observed to improve endothelial function and inflammatory biomarkers [12,13]. Moreover, data have shown that RSV improves glucose and glycated hemoglobin (HbA1c) and insulin profiles, thus preventing type 2 diabetes (T2D) and its cardiovascular complications. It also decreases inflammation, thus resulting in potential benefits for obesity [4,14,15]. 

All these effects, exerted on different systems and diseases, are made possible mainly through RSV’s interactions with several biological targets (e.g., sirtuin 1 (SIRT1), estrogen receptor α, different phosphodiesterases and cyclooxygenases) and its involvement in different key cellular pathways (such as PI3K/Akt/mTOR, AMPK/SIRT1/PGC-1α and NF-κB signaling), thus affecting antioxidant and anti-inflammatory processes; enhancing insulin sensitivity; regulating and improving lipid metabolism, glucose uptake and metabolism through the promotion GLUT4 expression and the translocation and activation of the SIRT1/AMPK signaling axis; protecting β-cells; and inducing autophagy [4,16,17,18].

Recent data also demonstrate that RSV modulates sphingolipid levels and signaling, with interesting consequences for the CM setting, as the accumulation of some ceramide species alters metabolic homeostasis and induces the onset and development of cardiometabolic diseases [7]. Sphingolipids are amphipathic lipids characterized by a sphingoid base backbone that is N-acylated with different fatty acid chains, with specialized regulatory roles in cellular metabolism depending on their characteristics (e.g., their fatty acyl chain lengths). Beyond their structural role, particularly in cell membranes, the amphipathic properties of these lipids and their subcellular distribution enable them to take part in a variety of key metabolic pathways (such as apoptosis, cell proliferation, oxidative stress and inflammation). As the modulation of specific ceramide levels might improve cardiometabolic health (e.g., through healthy dietary habits such as the Mediterranean diet, physical activity, weight loss or the development of pharmacological tools targeting CerS enzymes), these bioactive molecules are highly interesting for future health care strategies, such as those targeted towards the prevention and treatment of CM disease [7,19,20]. 

Accordingly, this review aims to discuss the available data on the effects of RSV on sphingolipid metabolism and signaling in terms of CM risk and disease, focusing, in particular, on oxidative stress/inflammatory-related aspects and the clinical implications of this relationship. 

## 2. Resveratrol: Classification and Metabolism in Brief

RSV (3,4′,5-trihydroxy-trans-stilbene) is a polyphenolic molecule that can be found in different plant species, such as blueberry, grapes, peanuts and cranberry, but red grapes (and, consequently, red wine) are the main source [21].

**Figure 1:**
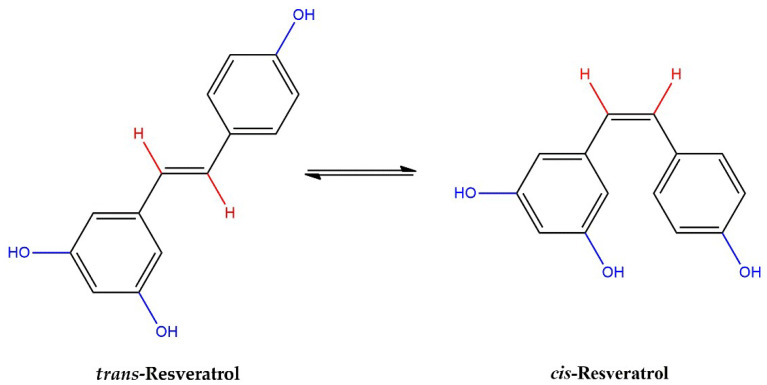
trans- and cis-RSV molecules.

The molecular structure of RSV includes hydroxyl groups, benzene rings and C–C double bonds. Thus, RSV exists as two geometric isomers, trans-RSV and cis-RSV, which are both present in red wine (Figure 1).

RSV can undergo degradation and chemical changes, being sensible to light, pH and temperature [21]. Thus, attempts have been made to improve the stability of RSV (e.g., its co-encapsulation with other bioactive molecules or the use of RSV nanosuspensions), aiming, in addition, to exploit this molecule for the development of new functional foods or nutraceutical supplements [4].

Moreover, efforts have been made to chemically synthesize RSV using different biosynthesis strategies, but in practice, many limitations hamper the application of such techniques for clinical purposes [21]. 

In the organism, RSV sustains considerable biotransformation, including conjugation (sulfation and glucuronidation), which mainly takes place in enterocytes, hydrolyzation and reduction at the gut level through microbiota activity, with the production of resveratrol derivatives (e.g., by acting on the double bond to generate dihydroresveratrol), and enterohepatic recirculation [22]. The polar metabolites of RSV are then excreted by the kidneys [22].

## 3. Resveratrol in Relation to Cardiometabolic Risk and Disease 

### 3.1. Preclinical Studies

RSV exerts antioxidant, anti-inflammatory, antifibrotic and cardiometabolic protective properties [23]. Moreover, it reduces platelet aggregation, enhances vasodilation (and nitric oxide bioavailability), reduces endothelial activation and modulates the metabolism of glucose and lipids. 

All these beneficial cardiometabolic RSV-related effects are mediated by different molecular targets, some of which have already been identified (e.g., the activation of sirtuin 1 and AMPK, as well as the inhibition of NfκB) (Table 1) [23]. Recently, a further possible mechanism that RSV uses to modulate endothelial function has been identified in the inhibition of the Pin1/Notch1 signaling pathway [24]. Nevertheless, it is known that RSV acts as an SIRT1 activator, decreasing vascular oxidative stress and preventing endothelial dysfunction [25]. In an in vitro model, the upregulation of SIRT1 appeared as a key factor for the activity of RSV in providing antiglycative/antioxidative defense and protection against high-glucose damage [26].

In two different in vitro models (rat aortic rings and human umbilical vein endothelial cells (HUVEC)), RSV was demonstrated to reduce both the acute high-glucose-induced endothelium-dependent relaxation damage induced by acetylcholine in phenylephrine-precontracted vessels, as well as cytotoxicity and oxidative stress, through a mechanism involving ROS scavenging, and was found to likely increase NO bioavailability [27]. The expression of antioxidant enzymes and endothelial-type nitric oxide synthase (eNOS) and reduction in the expression of inflammatory mediators are modulated by RSV through FoxO/SIRT1 activation and NRF2-dependent transcription [28]. Indeed, animal studies confirmed the beneficial effects of RSV supplementation on NO (circulating NO metabolites, eNOS expression, eNOS phosphorylation and eNOS uncoupling), generally in parallel to an improvement in blood pressure [29]. 

Moreover, RSV supplementation modulates the expression of different genes related to inflammation (INF-γ and TNF-α) and oxidative stress (heme oxygenase-1 and nitric oxide synthase) in the rat heart [30].

In rats, the administration of RSV (30 mg/kg·bw/daily for 10 weeks) reduced liver steatosis, oxidative stress and inflammation, improving the lipid profile as well as insulin sensitivity. It also reverted alterations in the hepatic mRNA expression levels of genes related to lipid metabolism and insulin signaling [31]. Moreover, RSV was shown to preserve β-cell function through the inhibition of the activity of phosphodiesterase, which hydrolyzes the phosphodiester bonds of cAMP and cGMP, thereby modulating various cellular signaling pathways [32]. Recent data also demonstrated that RSV reduces autophagy-mediated β-cell death via the inhibition of the CXCL16/ox-LDL pathway [33].

Other studies indicated that RSV reduces the apoptosis and modulates the paracrine function of cardiac microvascular endothelial cells under ischemia/reperfusion conditions [34]. In addition, RSV inhibits ox-LDL-induced endothelial injury (through the downregulation of circ_0091822 to upregulate miR-106b-5p-related TLR4) [35]. Ex vivo results on adipose tissue revealed that RSV inhibits adipogenesis through the activation of the AMPK-SIRT1-FOXO1 signaling pathway [36,37]. Moreover, RSV reduces the accumulation of triglycerides through the activation of PPARγ and SIRT1 [38,39].

Increasing evidence also highlights an important effect of RSV on the modulation of microbiota composition (type 2 diabetic animals and patients), similar to those induced by anti-diabetic drugs such as metformin, thus resulting in beneficial effects for T2D patients [40].

### 3.2. Clinical Studies

RSV supplementation had beneficial effects on some outcomes, such as blood pressure, the lipid profile, glycemic control and insulin resistance in T2D; the waist circumference in metabolic syndrome (MS); and body weight and inflammation markers in nonalcoholic fatty liver disease (NAFLD) [41]. In fact, it also improves insulin resistance and the glycolysis pathway, likely by modulating SIRT2, as evidenced using polycystic ovary syndrome granulosa cells [42]. Moreover, a meta-analysis of randomized controlled studies indicated that RSV is able to improve the lipid profile, reducing the TC, TG and LDL-C levels [11]. A meta-analysis of 25 studies (1171 participants, with 578 in the placebo group and 593 in the intervention group) demonstrated the benefits of RSV intake on lipid and glucose metabolism, with significant decreases in waist circumference, hemoglobin A1c, total cholesterol, low-density lipoprotein cholesterol and high-density lipoprotein cholesterol following RSV administration, exhibiting its major clinical value for obese and T2D patients [43]. Another recent meta-analysis of randomized controlled trials reported that RSV significantly enhances endothelial function, improving the flow-mediated dilation and intercellular adhesion molecule 1 (ICAM-1) levels [44].

An effect of resveratrol on mitochondrial function has been demonstrated in human muscle samples, which may constitute a beneficial effect in regard to cardiovascular disease (CVD) as well as the vascular and cardiac muscle, mitochondrial dysfunction being a general problem in CVD, in which different damaging processes can cause exhaustion that RSV may postpone [45]. 

RSV takes part in such a wide range of biological activities that it remains difficult to define its entire spectrum of actions. Moreover, many aspects need to be further evaluated, including the definition of an RSV threshold above which its effects are ensured, this also being the case for other variables that need to be taken into account (e.g., the durations of supplementation and the effects elicited, the RSV source (always being careful to avoid adverse effects derived from excessive alcohol consumption), age, the absorption and metabolism of RSV and other dietary components, but also individual genetic and gut microbiota characteristics) [30]. 

The recent available data essentially focus on the metabolic benefits of RSV in the context of obesity and T2D. In a meta-analysis including 11 randomized controlled trials (388 subjects), RSV significantly reduced fasting glucose, insulin, HbA1C and insulin resistance in T2D patients [46]. However, no significant effects of RSV on glycemic status were observed in the nondiabetic subjects [46]. Thus, further studies need to be planned based on the general population in order to evaluate the potential benefits of RSV in humans (e.g., the prevention of weight gain and metabolic dysfunction). 

The combination of RSV with other substances may also be more effective in the treatment or prevention of cardiometabolic diseases, this being a strategy that is currently under evaluation based on subjects with metabolic syndrome, T2D or obesity (e.g., hesperetin; δ-Tocotrienol; a combination of curcumin, RSV, zinc, magnesium, selenium and vitamin D) [47,48,49,50].

Moreover, synthetic nano-systems or biologically derived carriers may incapsulate RSV, enhancing its physicochemical properties and targeted delivery, thus opening up a wide range of applications, including the CM setting [51].

**Table 1:** Main resveratrol pathways and effects involved in cardiometabolic benefits.

Pathways and Effects	References
SIRT1 activation, AMPK activation, Pin1/Notch1 inhibition	[24,36,37,42]
Modulation of the expression of antioxidant genes	[28]
Anti-inflammatory effects (e.g., NFκB inhibition)	[23,28,30,31]
Improvement of endothelial function, nitric oxide bioavailability, protection against LDL-ox	[25,29,35,43]
Improvement of insulin sensibility, reduction in insulin resistance	[31,44]
Glucose uptake and improvement of glucose metabolism	[4,16,18]
Prevention of β-cell dysfunction and death	[32,33]
Adipogenesis inhibition (AMPK-SIRT1-FOXO1)	[36,37]
Improvement of lipid metabolism	[11]
Improvement of blood pressure	[29]
Favorable microbiota composition	[40]

## 4. Sphingolipid: Functional Active Members and Metabolism 

**Figure 2:**
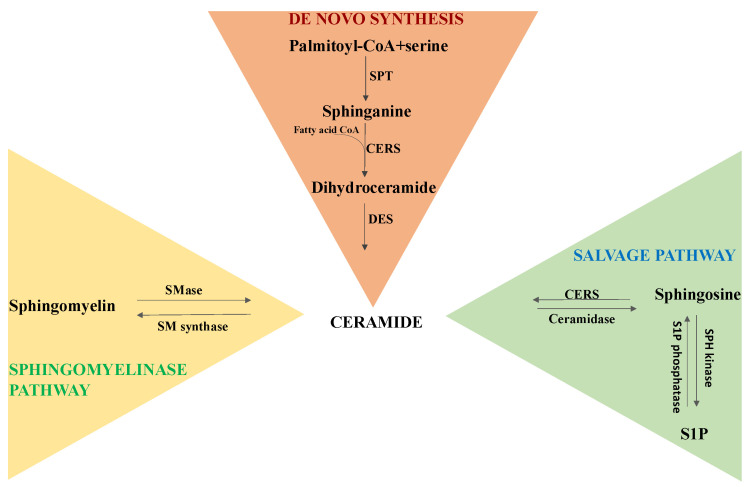
Synthesis of ceramides.

The most functionally active members of the SL family are sphingomyelin (SM, which is formed of a phosphocholine head group, a sphingosine and a fatty acid), ceramides (CER, consisting of a sphingoid base linked to a fatty acid through an amide bond), sphingosine (SPH, an 18-carbon amino alcohol with an unsaturated hydrocarbon chain, representing the sphingolipid backbone) and sphingosine-1-phosphate (S1P, a sphingolipid with a phospho-group attached to the carbon in position 1) [52]. Ceramides, which act as the structural unit for the biosynthesis of more complex SL species, are mainly synthesized via three metabolic pathways (Figure 2):

De novo synthesis takes place in the cytosolic layer of the endoplasmic reticulum. The rate-limiting enzyme of this pathway is serine palmitoyl-CoA transferase (SPT), a heterodimer formed of two subunits that catalyzes the condensation of palmitoyl-CoA and serine, generating 3-keto-dihydrosphingosine. 3-Keto-dihydrosphingosine reductase produces sphinganine, while dihydroceramides are generated by different isoforms of ceramide synthase (CERS) through the incorporation of acyl-CoA of different chain lengths. Ceramides are then formed by a dihydroceramide desaturase that introduces a double bond in positions 4–5 (trans-configuration) of dihydroceramide. Newly biosynthesized ceramides are transported into the Golgi apparatus, where they can be metabolized into complex sphingolipids (e.g., sphingomyelin, glucosylceramides and gangliosides) and can also be phosphorylated by the ceramide kinase (CerK) to generate ceramide-1-phosphate (C1P) [20]. Then, the sphingomyelinase pathway: ceramide is also generated through the hydrolysis of sphingomyelin by neutral or acidic sphingomyelinases (SMase) [20]. Salvage pathways: this process takes place in endo-lysosomes. Ceramides are produced from sphingosine through ceramide synthase (CERS), while the ceramidase enzyme catalyzes the hydrolysis of ceramide and leads to the production of sphingosine and a fatty acid. Then, sphingosine kinase (SPH kinase) generates sphingosine-1-phosphate (S1P), and sphingosine phosphatase (S1P phosphatase) can generate sphingosine [20].

## 5. Sphingolipids and Cardiometabolic Risk

CER acts as a second messenger, regulating many cellular processes. In particular, several studies showed a relationship between CER and apoptosis in the pathogenesis of many diseases, such as T2D, stroke and myocardial infarction [53,54,55]. Indeed, CER could induce endothelial cell apoptosis [56], causing plaque erosion with complications related to the atherosclerotic process. Moreover, ceramide accumulation may denote many adverse oxidative stress-related events, such as the inhibition of Akt to abrogate insulin signaling, excessive lipid storage through the inhibition of the hormone-sensitive lipase and mitochondrial function, while, on the other hand, increasing oxidative stress and the release of pro-inflammatory cytokines [19].

The exposure of LDL to bacterial SMase in vitro induces the formation of LDL aggregates that can be retained by the extracellular matrix and can stimulate macrophage foam cell formation [57]. The SMase-induced aggregation of LDL in vitro requires the active SMase enzyme and is mediated via a high particle ceramide content. When the ceramide content of lesional LDL (from human atherosclerotic plaque) was assayed, it was found to be 10–50-fold enriched compared with plasma LDL ceramide. Moreover, the ceramide was found exclusively in the lesional LDL that was aggregated; unaggregated lesional LDL, which accounted for 20–25% of the lesional material, resulted in reduced ceramide [58]. 

S1P is involved in proliferation, cell growth, survival, inflammation, angiogenesis and resistance to apoptotic cell death. The effect of S1P is mediated by the S1P receptors connected with different types of G-proteins, being receptors that induce the activation of intracellular signaling pathways, so that S1P behaves not only as an intracellular messenger but also as an extracellular player, binding to the plasma membrane G-protein-coupled receptor [52,59]. There are five S1P receptors, identified as S1Pr1–S1Pr5, that are ubiquitously expressed in cells, including cardiomyocytes [60]. Evidence supports a cardioprotective role of S1P through the S1PRs signaling pathway [61]. Moreover, S1P inhibits oxidative stress and inflammatory responses [62] and positively modulates mitochondrial function, protecting cardiomyocytes from hypoxia/reoxygenation injury (the JAK-STAT pathway and Akt/Gsk3β phosphorylation are likely involved in this event) [63,64].

### 5.1. Ceramides: Human Studies

The relationship between ceramides and coronary artery disease (CAD) has been demonstrated in many studies (clinical trials, case–control and cohort studies) [65,66,67,68]. However, among all the species detected in plasma, only some ceramides appear to be associated with the prediction of acute myocardial infarction (AMI) and CV death in healthy subjects. Those species generally found to be more involved in this relationship are Cer (d18:1/16:0), Cer (d18:1/18:0), Cer (d18:1/20:0) and Cer (d18:1/24:1), and their ratios with Cer (d18:1/24:0) are also significant [67]. We also have evidence showing that distinct ceramide species are associated with CV risk factors, inflammatory parameters and disease severity in AMI [65]. 

In patients with stable CAD or in secondary prevention after AMI, the same ceramide species were able to predict the risk of adverse outcomes [69]. In another study, where CAD patients were divided into four groups according to the severity of coronary artery stenosis, four plasma ceramide species (Cer (d18:1/16:0), Cer (d18:1/18:0), Cer (d18:1/24:1), Cer (d18:1/24:0)) were analyzed. The results showed that, based on logistic regression analysis, the ratio of Cer (d18:1/24:1) to Cer (d18:1/24:0) was significantly increased in subjects with stenosis > 50% compared to those with stenosis < 50%. After multiple logistic regression analysis, adjusted for clinical risk factors, the severity of stenosis was associated with a high ratio of Cer (d18:1/24:1) to Cer (d18:1/24:0), female gender, HbA1c% and AMI [69]. CER has been strongly implicated in T2D, since it plays a role in insulin resistance [70]. Recently, no relationship between ceramide levels and stress hyperglycemia (a common status of transient elevation of blood glycemia due to stress caused by illness) has been detected, but rather specific species were elevated in T2D in the context of AMI [71].

In the Strong Heart Family Study, sphingolipids were evaluated with both baseline and follow-up analyses of plasma insulin, HOMA of insulin resistance (HOMA-IR) and HOMA of β-cell function (HOMA-B) after adjustment for risk factors. In 2086 participants without T2D, higher levels of plasma Cer (d18:1/16:0), Cer (d18:1/18:0), Cer (d18:1/20:0) or Cer (d18:1/22:0) were associated with higher plasma insulin and higher HOMA-IR levels at baseline and at follow-up an average of 5.4 years later. In particular, the relationship between species carrying 18:0, 20:0, 22:0 or 24:0 and insulin was modified by BMI [72]. 

### 5.2. S1P: Human Studies 

S1P and its G-protein-coupled receptor play an important role in the cardiovascular system. In 1995, Bünemann et al. demonstrated the existence of S1P receptors in the heart [73]: since then, several other studies have demonstrated in the expression of S1P1, S1P2 and S1P3 receptors adult mammalian hearts [74]. In atherosclerotic patients with peripheral artery disease or carotid stenosis, the levels of blood S1P were lower than those observed in healthy subjects; moreover, serum-S1P increased after treatment (independent of the atherosclerotic site or procedure type, i.e., open or endovascular surgery) [75]. In patients with CAD, the increase in the serum level of S1P was directly related to the severity of stenosis and more predictive of obstructive CAD compared to the formal risk factors (i.e., sex, age, family history of CAD and hypertension) based on multivariate logistic regression analysis [76]. In patients with AMI (undergoing primary percutaneous coronary intervention, PCI), the S1P blood levels were higher compared to those with stable CAD, showing a gradual decrease 48 h after PCI in the AMI group, without significant variation among the stable CAD patients [77]. Knowledge of these distinct behaviors in two different clinical situations is important, especially in view of the fact that pharmacological agents are available (e.g., fingolimod or amiseliod, S1P receptor modulators), which may also help to develop promising therapeutic strategies in the cardiovascular field [78]. 

Regarding T2D and insulin resistance in humans, S1P is generally found to be increased in patients with obesity and insulin resistance [79], although some data did not demonstrate any change or blood S1P decrement with the development of insulin resistance and T2D [80,81,82]. This behavior, which may appear contradictory, can be explained by different reasons: for example, the S1P concentration is significantly reduced in parallel to kidney dysfunction in T2D; moreover, it appears to be modulated differently in males and females under the effects of sexual hormones (e.g., estradiol stimulates SPH kinase and the release of S1P) [83,84,85]. It is noteworthy that S1P is bound to different transport proteins (e.g., ApoM-bound S1P or albumin-bound S1P), which may drive different biological functions [86,87,88].

## 6. Effects of Resveratrol on Sphingolipid Metabolism: Possible Implications on the Clinical Level

The membrane interaction of RSV has been highlighted, particularly the ability of this molecule to affect the lipid bilayers (e.g., transmembrane proteins) and enzymes whose activity is related to the structural membrane organization and the presence/absence of these domains [89]. In RSV-treated hepatocytes isolated from old rats, plasma membranes were isolated from controls, and the lipid composition and metabolism were analyzed [90]. The results showed that RSV treatment significantly reduced the ceramide content in the plasma membranes of senescent hepatocytes by inhibiting SMase activity. Moreover, the accumulation of sphingomyelin, a lipid acting as a natural membrane antioxidant, was mainly related to the reduced SM hydrolysis, induced by SMase in the RSV-treated aged cells [90]. RSV has a different action on sphingolipid metabolism if the treated cells are cancerous: indeed, in cancer cells, Scarlatti et al. reported that RSV induced a 50% increase in the activity of SPT and a 4.2-fold activation of SMase, thus contributing together to the accumulation of endogenous ceramides in breast cancer cells [91]. During the RSV treatment of A549 lung adenocarcinoma cells, there was an increase in CER and sphingosine and a decrease in sphingomyelin and S1P. In these cells, the activity and expression of CER synthase 6 were upregulated, demonstrating that CER was accumulated through de novo synthesis [92]. Other data also indicate that RSV induced apoptosis and decreased SPH kinase activity and the S1P levels while increasing the level of ceramides in a dose-dependent manner in a leukemia cell line (K562) [93].

RSV also acts on the protein sirtuins (e.g., SIRT1), explaining its different biological effects, including its cardioprotective and anti-inflammatory effects [17], the regulation of hepatic lipid metabolism and oxidative stress and the inhibition of hepatic inflammation [94,95]. In HUVEC treated with oxidized LDL (ox-LDL), the effect of the protective mechanism of RSV on high-fat-induced endothelial dysfunction was investigated. While ox-LDL induced endothelial cell apoptosis and proliferation arrest, treatment with RSV reduced these phenomena. Moreover, RSV prevented ox-LDL-mediated mitochondrial respiratory complex inactivation through the upregulation of BCL2-interacting-protein-3 (Bnip3)-related mitophagy, sustaining mitochondrial membrane potential and favoring endothelial cell survival [96].

Taken together, these findings suggest that RSV administration inhibits SPH kinase and decreases SM and S1P, reducing downstream effects and increasing ceramide levels [97,98,99]. Moreover, on the pulmonary level, RSV inhibits pulmonary arterial remodeling by suppressing SPH-kinase-mediated NF-κB activation, with benefits for pulmonary arterial smooth muscle cell proliferation and pulmonary vessel muscularization [100].

RSV’s capacity to inhibit SPH kinase activity might reduce cardio protection, provided by S1P associated with the HDL, and S1P-dependent activation of pro-survival signaling through pathways such as the PI3K/Akt and MAPK pathways [101,102,103,104,105]. However, S1P also induces cardiac hypertrophy, decreases the heart rate, reduces ventricular contraction and exerts artery vasoconstriction, adverse effects that can theoretically be improved by inhibiting SPH kinase activity or expression with resveratrol [106,107,108,109].

It is clear that the effects of RSV, through the modulation of sphingolipid activity and signaling on the cardiovascular level, are difficult to assess. In fact, the number of effects exerted by S1P in the cardiovascular system makes it difficult to evaluate the benefit of reducing S1P levels without avoiding side effects. Moreover, the increase in ceramides is associated with pro- or anti-atherogenic effects, as these molecules can induce protective or adverse consequences depending on the length of their sphingoid bases, biosynthetic pathways, activated signaling cascades and intracellular environment [110]. It is also important to consider the possibility that a change in a target ceramide might lead to a compensatory modification in the levels of other ceramides or downstream sphingolipid species with cytotoxic properties. Thus, it is critical to evaluate the overall types of ceramides before drawing conclusions about changes in a specific ceramide species.

Currently, new proposed pharmacological tools, such as transporter modulators, biased agonists of S1PRs, S1P chaperones and ligand neutralization strategies, are under study [111]. Nonetheless, the use of fingolimod (a sphingosine-1-phosphate receptor modulator) in autoimmune disease may induce opposite effects on the cardiovascular level, such as bradycardia, vasodilation and an increase in endothelial cell barriers, protecting against vascular leakage (due to fingolimod’s action as a full S1PR agonist), followed by vasoconstriction and macular edema (due to the S1PR antagonist’s behavior after the downregulation of the S1PR subtype 1 on the cell surface under persistent fingolimod administration), thus retaining possible adverse repercussions for the CV system [112]. Moreover, it must be considered that the effects of RSV on sphingolipid metabolism on the CV level are likely concentration- and time-dependent [113].

Interestingly, it has been observed that concentrations of RSV of up to 50 µM increase sphingosine, ceramide and S1P levels in human skin cells, with no significant increase in apoptosis, whereas higher concentrations (100 µM) significantly reduce cell viability [114]. Thus, it is possible, especially at high concentrations (>50 µM), that RSV increases pro-apoptotic ceramide and reduces pro-survival S1P levels, which suppress cell growth and induce apoptosis.

Moreover, a further understanding of the long-term effects of RSV on sphingolipid metabolism is also needed, especially with regard to the effects focused on the CV system, because currently, the majority of RSV-mediated mechanisms affecting sphingolipid metabolism, being strongly linked to oxidative stress and inflammation, have been obtained in the field of cancer [115]. RSV acts through multiple mechanisms, modulating different aspects of carcinogenesis, angiogenesis and metastasis, suggesting possibilities for the combined use of this polyphenol with conventional agents for the development of complex therapeutic strategies [116]. Concerning sphingolipid levels, RSV alters their balance towards an increase in CER levels through the enhancement of CER levels and the induction of apoptosis, as observed in colon, breast and prostate cancer cells, as well as the downregulation of SK1 expression and activity, as evidenced in prostate cancer cells [113,117,118,119].

In particular, it has been observed that RSV and ceramides converge in an endocytosis-requiring, ERK1/2-dependent signal transduction pathway, and the induction of COX expression has been identified as a critical molecular step before the subsequent p53-dependent apoptosis [120]. 

Other recent data demonstrated that RSV triggers anti-proliferative cellular effects by inhibiting ceramide catabolism, and in view of this behavior, the authors suggested its possible evaluation as a chemopreventive agent (always after careful consideration and the acquisition of additional knowledge about the crosstalk between resveratrol and the ceramide catabolism pathway) [121]. In this context, in human lung adenocarcinoma A549 cells, incubation with RSV for 24 h at a concentration of 100 µM significantly inhibited SphK activity and decreased S1P levels, decreasing ceramide levels [92]. However, in a previous study, the same authors reported that RSV induced an increase in sphingomyelin and a decrease in CER in senescent rat hepatocytes [90]. These controversial results may find an explanation in the different types of cells considered: normal hepatocytes isolated from healthy aged animals versus cancer cells, meaning that the final RSV effects may vary according to the target considered. 

Thus, whether these many observations might open up the possibility of the use of RSV in sequence or combination with conventional therapy for chemoprevention and cancer treatment, there is still much work to be done in order to better understand the complex actions of RSV and different mechanisms and effects triggered by the modulation of RSV on sphingolipid metabolism in cancer. In fact, the anti-CVD and anti-cancer effects observed are often due to a dose-dependent effect of RSV, whereas further, future trials are expected to investigate dose-dependent effects and the possible toxicity of this molecule [122]. Accordingly, a recent systematic review and meta-analysis of randomized controlled trials investigating the effect of RSV on liver biomarkers in adult participants demonstrated that high-dose RSV should be administered with caution [123]. In fact, high-doses of RSV supplementation (>1000 mg/day) appeared to increase alkaline phosphatase concentrations, while alanine aminotransferase was also increased in older subjects following RSV supplementation. 

As RSV is highly important for sphingolipids, which are most prominent in the neuronal tissues, RSV has attracted attention as a promising therapeutic agent that may potentially be useful in preventing and treating neurological disease (e.g., Alzheimer’s disease, Parkinson’s disease) [124]. In fact, RSV retains its neuroprotective effects through its antioxidative capacities and protects mitochondrial activity [125]. RSV also modulates key genes involved in antioxidant enzyme activity and cell survival [125]. In particular, RSV-induced SIRT1 activation, which can protect against the adverse consequences of oxidative stress, facilitating neuroprotection and promoting neurogenesis in the hippocampus of the adult brain, is becoming a critical starting point for the development of new therapeutic options in this field [124,126]. 

With regard to inflammatory conditions, RSV is able to block NF-kB activation induced by ceramides and reduce reactive oxygen intermediate generation and lipid peroxidation in in vitro models [127]. Moreover, recent data indicate that RSV protects against skin inflammation through multiple mechanisms of action, including decreased activation of the S1P-producing enzyme SPH kinase, transcription factors (the signal transducer and activator of transcription 3, Stat3) and NFκBp65, involved in chemokine production in mice [128].

## 7. Conclusions

RSV exhibits a number of pleiotropic actions, being generally beneficial in various pathophysiological conditions and targeting key regulator pathways. The RSV-mediated modulation of sphingolipid metabolism and signaling may represent one key mechanism through which RSV exerts its effects, including that on oxidative stress balance. Thus, RSV can aid in the prevention of, and be considered as an adjuvant treatment for, CM disease, whereas sphingolipid may act as an interesting therapeutic target. However, many aspects of this molecule are still unknown and unclear. One of the main limits of the use of RSV, among others, is its bioavailability; thus, the study of novel drug delivery systems may help to overcome this difficulty. Remarkably, although this molecule represents an exciting adjuvant tool to be further studied as a possibility for the development of complex therapeutic strategies that may be used to prevent and address CM risk and disease, its complexity, together with its wide range of actions, are yet to be completely clarified, especially with regard to its differential actions on sphingolipids (with beneficial or even possibly adverse effects) or differential dose-related effects on oxidative stress and inflammation.
